# Paracetamol versus ibuprofen for early postpartum pain control: a randomized controlled trial

**DOI:** 10.1007/s00404-024-07797-4

**Published:** 2024-11-05

**Authors:** Shai Ram, Dotan Madar, Hila Shalev Ram, Goni Peleg, Yotam Lior, Ayelet Greenfeld, Gala Yakov, Yariv Yogev, Sharon Maslovitz

**Affiliations:** 1 Lis Maternity and Women’s Hospital, 6 Weitzman St, 6423906 Tel Aviv, Israel; 2 Division of Anesthesia, Intensive Care, and Pain Medicine, Tel Aviv Sourasky Medical Center, Tel Aviv, Israel; 3 Department of Obstetrics and Gynecology, Meir Medical Center, Kfar Saba, Israel; 4 Faculty of Medicine, Tel Aviv University, Tel Aviv, Israel

**Keywords:** Postpartum pain, Non-opioid treatments, Ibuprofen, Paracetamol, Pain relief strategies after vaginal delivery

## Abstract

**Introduction:**

To evaluate the effectiveness of paracetamol and ibuprofen as non-opioid treatments for postpartum pain control after vaginal delivery.

**Materials and methods:**

This randomized controlled study at a university-affiliated medical center involved parturient who received blindly oral tablets of either 1000 mg of paracetamol or 400 mg of ibuprofen, post-vaginal birth. Pain levels were assessed using a numeric rating scale (NRS) at four time points: before treatment, and 1, 4, and 6 h post-treatment (T0, T1, T4, and T6, respectively). We also compared the need for additional analgesia, breastfeeding initiation, mobilization, and urination following the delivery between the groups. To ensure statistical power, the study was designed to detect differences of one point on the NRS with at least 37 women per group.

**Results:**

A total of 107 women participated, including paracetamol (n = 52) and ibuprofen (n = 55) groups. Demographics and perinatal outcomes were similar across groups. No significant differences were found in the interval between delivery and request for pain control (8 ± 6–10.5 and 11 ± 6–16 h for the paracetamol and the ibuprofen, respectively, *P* = .13). Pain levels on the NRS were similar for both groups at all intervals. There were also no group differences in the time to the initiation of breastfeeding, mobilization, urination, or the need for additional analgesia.

**Conclusion:**

Both, paracetamol and ibuprofen, can be considered equivalent and effective non-opioid alternatives for postpartum pain control.

**Registry at clinicaltrials.gov:**

(NCT04653506), https://register.clinicaltrials.gov/prs/beta/studies/S000AFOR00000066/recordSummary.

## What does this study add to the clinical work

**Table:** 

Paracetamol and ibuprofen provide comparable immediate postpartum analgesia, presenting possible non-opioid alternative for pain management.	

## Introduction

Postpartum pain is common, with 75–97% of women reporting perineal pain within one day of giving birth, and as high as 90% of those women reporting that the pain impedes their ability to carry out daily activities and care for their newborns [1]. Uncontrolled postpartum pain has been associated with significant adverse outcomes, such as difficulties in emotion regulation, maternal depression, chronic pain, and increased reliance upon opioid medications [2–5]. Approximately 1.7% of women who underwent vaginal delivery and 2.2% of those who underwent a cesarean section reportedly developed a persistent pattern of opioid usage [3]. Long-term use of opioid medications is associated with several disadvantages, including substance dependency [15] and breastfeeding that can be affected by the transfer of opioids through breastfeeding [6], potentially leading to undesirable effects on the newborn, such as drowsiness. These concerns have led to the search for equally effective non-opioid strategies for postpartum pain relief to replace opioid use and enhance recovery [1, 7, 8].

While numerous analgesic regimens are commonly practiced in the clinical setting, scientific literature on comparisons of their efficacy is lacking. Paracetamol and members of the NSAIDs family, such as ibuprofen, are among those agents used in common practice.

A systematic Cochrane meta-analysis [9] assessed the efficacy of paracetamol treatment compared with placebo for perineal pain relief after childbirth. Although the studies included in the review found paracetamol to be more effective than placebo in alleviating pain, another study [10] demonstrated the superiority of non-steroidal anti-inflammatory drugs (NSAIDs) over a placebo in relieving pain following vaginal delivery. However, there was no significant difference between treatment nor any significant difference in pain relief originating from immediate postpartum uterine contractions between paracetamol and placebo. These findings are also supported by a meta-analysis [9]. The conclusions that were reached in that review, however, were based upon a single study with a small sample size (48 cases). A Cochrane metanalysis [11] indicated that NSAIDs were more effective than placebo for perineal pain relief after vaginal delivery at both 4 and 6 h after administration, although the quality of the evidence was considered to be low. Additionally, that meta-analysis found that NSAIDs were more effective than paracetamol at 4 h but not significantly different at 6 h. As in earlier meta-analyses, the studies included in that review were outdated, and the authors acknowledged potential biases in the results, highlighting the need for updated non-biased studies.

It has not been established which non-opioid interventions are effective for specific types or locations of pain (e.g., perineal pain, uterine contractions, back pain), nor have the factors of general reduction of postpartum pain, the time interval between childbirth and the need for pain relief, and the potential impact of pain control on breastfeeding ability.

Thus, we aimed to compare single administration of non-opioid medications, specifically paracetamol (1000 mg) and ibuprofen (400 mg) in terms of their effectiveness in relieving general postpartum pain across different time frames. We also aimed to assess the association between postpartum pain relief with the time to the initiation of breastfeeding, mobility and spontaneous urination, and the need for additional pain relief.

## Materials and methods

We conducted a double-blind, randomized controlled interventional study at a single university-affiliated medical center. This study was approved by the local Institutional Review Board (IRB number 036820-TLV) and registered at clinicaltrials.gov NCT04653506. The study started on 22/11/2020 and ended on 18/02/2024.

The study included a cohort of consecutive women who underwent spontaneous vaginal delivery between February 2021 and June 2022 and provided informed consent to participate. Excluded were women who had undergone a caesarean section, had a known sensitivity to any of the study medications, were classified as high-risk pregnancies (such as preeclampsia or gestational or pre-gestational diabetes), those with chronic pain or fibromyalgia and those experiencing third- or fourth-degree perineal tears. Women who had received pain relief prior to being approached for study participation were not initially recruited. It should be noted that in the department where the study was conducted, epidural analgesia was discontinued immediately after delivery. Women did not routinely receive pain relief post-delivery unless they requested it, adhering to a pain management protocol that commenced with non-opioid medications.

Women who enrolled in the study, recruited as close to the time of delivery as possible, met the inclusion criteria and had not been treated with pain relief medications prior to their recruitment.

## Methods

When women recruited for the study requested analgesia, they were randomly and double blindly assigned to one of two treatment groups by means of pre-prepared envelopes containing tablets of ibuprofen 400 mg or paracetamol 1000 mg. The doses selected for this study align with the standard pain relief protocols of our department, and are consistent with those utilized in previous studies [9–13]. No placebos were administered as part of the study protocol. Pain assessment following the administration of a tablet was by the NRS index at 4 time points: at treatment onset, 1-, 4-, and 6-h post-treatment (T0, T1, T4, and T6, respectively). Participants were also asked to indicate the location of their most intense pain requiring pain relief, such as the vaginal area/sutures, abdomen/uterus, back, or another location.

If a study participant still experienced pain after receiving double-blinded analgesia during the 6-h follow-up period post-administration, additional pain relief was provided using 1 g of dipyrone. If a participant was sensitive to dipyrone, a research team member would identify the medication administered under the double-blind mechanism, and the participant would then be treated with the alternate medication she had not received (for instance, if the participant received paracetamol under double-blind conditions, she would be treated with ibuprofen). If the additional pain relief was insufficient, participants could receive supplementary opioid analgesia. In any case, participants were instructed not to self-administer any additional pain relief during the six hours follow-up period. If additional analgesia was provided by the department staff, it was recorded, as well as the ability to initiate breastfeeding, duration between childbirth and the woman’s ability to mobilize and urinate.

Data on the women’s age at the index childbirth, gestational week, birth number, pregestational body mass index, as well as delivery details, including the administration of epidural anesthesia, vacuum-assisted delivery, postpartum hemorrhage (PPH), manual removal of placenta, and the presence and degree of tears were extracted from electronic medical records. PPH was defined based on the delivery report. The diagnosis was based on either the subjective impression of more than usual bleeding or objective criteria such as a decrease in hemoglobin levels of more than 3 g, hemodynamic changes, or the need for a blood transfusion.

The NRS score relative to T0 was calculated by dividing the scores at T1, T4, and T6 by the NRS score at time T0 and multiplied by 100 (to obtain percentage). This calculation provided a relative measure of pain intensity based upon the initial NRS score. We also compared the ability to initiate breastfeeding between the 2 groups and expressed it as a success rate within the designated timeframe (up to 6 h from T0). The duration until ambulation and urination after delivery were also compared and reported as the median time with the interquartile range [IQR] in hours. Finally, we calculated the percentage of women who required additional pain relief following the first dose (T0) at any time during the study period.

### Statistical analyses

For the purpose of calculating statistical power for this study, we designed it to be sufficiently powered to detect clinically significant differences of up to one point on the NRS with a minimum of 37 women in each group. The calculation of the statistical power was based on cohort study of 37 women treated with paracetamol compared to 36 women treated with ibuprofen demonstrated a difference in the relief of postpartum episiotomy pain of 50% between the groups [14]. Although no differences were found in a Cochrane study that compared treatments for uterine cramping [13], another Cochrane study that compared ibuprofen with paracetamol for treating perineal pain demonstrated an advantage of more than 50% for the former within 4 h post-administration [11].

Data collected in this study were documented on summary tables. The preferred method of analysis for continuous variables was parametric by means of the student t-test. The non-parametric Mann–Whitney test was used if parametric assumptions could not be satisfied, even after data transformation attempts. Parametric model assumptions were assessed using Normal-plot or Shapiro–Wilks statistics for verification of normality and Levene’s test for verification of homogeneity of variances. Categorical variables were tested with the Pearson’s χ2 test for contingency tables or the Fisher Exact test, as appropriate. All statistical tests and/or confidence intervals were performed at α = 0.05 (2-sided). All *P* values were rounded to 2 decimal places. The data were analyzed using IBM SPSS Statistics software, version 29.

## Results

A total of 166 women were recruited. Of these, 18 women did not require any pain relief during their stay in the department, 28 women used pain relief methods not adhering to the study protocol, either without double-blind conditions or independently and 13 women declined to participate after having already signed the consent form. Overall, 107 women randomized and participated in the study: 52 in the paracetamol group and 55 in the ibuprofen group.

The demographic characteristics and labor and delivery data were comparable for the two studied groups (Table 1). Assessment of the effectiveness of either analgesia revealed that there were no significant differences between the time since delivery to the request for analgesia (8 [6–10.5] for paracetamol vs. 11 [6–16] hours for ibuprofen, *P* = 0.13) (Table 2), nor in their NRS scores at any of the measured time points. Additionally, no significant difference was found in the time since delivery to the request for analgesia, even when a subgroup analysis was conducted for the population of women who delivered with epidural analgesia compared to those without (11.1 [7–15] hours vs. 10.2 [7–14.5] hours, respectively, *P* = 0.18).In addition, there were no significant group differences in their NRS scores, NRS scores at T0, or in the need for additional analgesia at the selected time points (T1, T4, or T6). However, a higher percentage of women in the ibuprofen group reported experiencing abdominal/uterine cramping as being the most painful location (89% compared to 59.2% for the paracetamol group, *P* = 0.002).

**Table 1:** Demographic and labor and delivery characteristics

Characteristic	Total cohort (*n* = 107)	Paracetamol (*n* = 52)	Ibuprofen (*n* = 55)	*P* value
Age, mean (SD), y	32.94 (4.2)	33.02 (4.35)	32.86 (4.1)	0.84
Gestational age mean (SD), wks	39.58 (1.05)	39.67 (1.14)	39.5 (0.96)	0.41
Parity (IQR)	2 (1–2)	2 (1–2)	2 (1–2)	0.74
Pregestational BMI, median (IQR)	22.31 (19.7–25.7)	22.61 (20.3–25.7)	22.31 (19.3–25.9)	0.59
Vacuum extraction	9 (8.4)	4 (7.7)	5 (9.1)	1
Epidural	88 (82.2)	43 (82.6)	45 (81.8)	0.89
No analgesia	19 (17.7)	10 (19.2)	9 (16.3)	0.7
PPH/revisio uteri	4 (3.7)	0 (0)	4 (7.3)	0.12
No tears	40 (37.4)	19 (36.5)	21 (38.2)	0.86
Labial or 1st degree tear	34 (31.8)	15 (28.8)	19 (34.5)	0.53
Episiotomy or 2nd degree tear	42 (39.3)	20 (38.5)	22 (40)	0.87

Values are given *n* (%) unless indicated otherwise

*BMI* body mass index, *PPH* post-partum hemorrhage, *SD* standard deviation

**Table 2:** Effectiveness of analgesia

Variable	Total cohort (*n* = 107)	Paracetamol (*n* = 52)	Ibuprofen (*n* = 55)	*P* value
Time delivery to analgesia request, median (IQR), hr	9 (6–12.5)	8 (6–10.5)	11 (6–16)	0.13
*T0*				
NRS, median (IQR)	6 (5–7)	6 (5–7)	6 (5–6)	0.3
Location, *n* (%)	27 (35.5)	17 (37.8)	10 (32.3)	0.62
Sutures/vagina	69 (72.6)	29 (59.2)	40 (87)	0.002
Abdomen/uterus	15 (20.5)	8 (19)	7 (22.6)	0.71
Back	3 (4.3)	0 (0)	3 (10.3)	0.07
*Other*				
*T1*				
NRS, median (IQR)	1.5 (0–4)	1 (0–4)	2 (0–3)	0.72
NRS relative to T0, median (IQR), %	32.67 (0–66.6)	20 (0–66.7)	33 (0–60)	0.68
Request for further analgesia, *n* (%)	14 (13.1)	8 (15.4)	6 (10.9)	0.49
*T4*				
NRS, median (IQR)	1 (0–5)	2 (0–5)	0.5 (0–5)	0.23
NRS relative to T0, median (IQR), %	20 (0–71.4)	33.3 (0–83.3)	8.3 (0–66.7)	0.2
Request for further analgesia, *n* (%)	25 (23.3)	12 (23.1)	13 (23.6)	0.88
*T6*				
NRS, median (IQR)	1 (0–3)	1 (0–4)	0.5 (0–3)	0.16
NRS relative to T0, median (IQR), %	16.67 (0–52.5)	20 (0–66.7)	6.3 (0–42.5)	0.16
Request for further analgesia, *n* (%)	19 (17.8)	10 (19.2)	9 (16.4)	0.7

*IQR* interquartile range, *NRS* numeric rating scale

There was only one woman who required opioid pain relief during the 6-h follow-up period. In this case, the woman was treated under double-blind conditions with ibuprofen. At T1 she experienced insufficient pain relief, necessitating the use of dipyrone. At T4, she still reported pain and therefore required opioid analgesia.

The percentage of women who initiate breastfeeding was comparable among women in both groups (86% for the entire population, 82.6% for the paracetamol group, and 88.9% for the ibuprofen group, *P* = 0.37), the initiation of mobilization (median 5 [4–6.5] hours, 5 [4–6] hours, and 5 [4–7] hours, respectively, *P* = 0.48), the time to first spontaneous urination (median 5 [4–7] hours, 5 [4–8] hours, and 5 [4–6] hours, respectively, *P* = 0.96), and the need for additional analgesia (46.7%, 44.2%, and 49.1%, respectively, *P* = 0.62) (Table 3).

**Table 3:** Comparative analysis of postpartum outcomes

Variable	Total cohort (*n* = 107)	Paracetamol (*n* = 52)	Ibuprofen (*n* = 55)	*P* value
Breastfeeding, *n* (%)	86 (86)	38 (82.6)	48 (88.9)	0.37
Time to mobilization, median (IQR), hr	5 (4–6.5)	5 (4–6)	5 (4–7)	0.48
Time to spontaneous urination, median (IQR), hr	5 (4–7)	5 (4–8)	5 (4–6)	0.96
Time for the need of additional analgesia, *n* (%)	50 (46.7)	23 (44.2)	27 (49.1)	0.62

*IQR* interquartile range

**Fig. 1:**
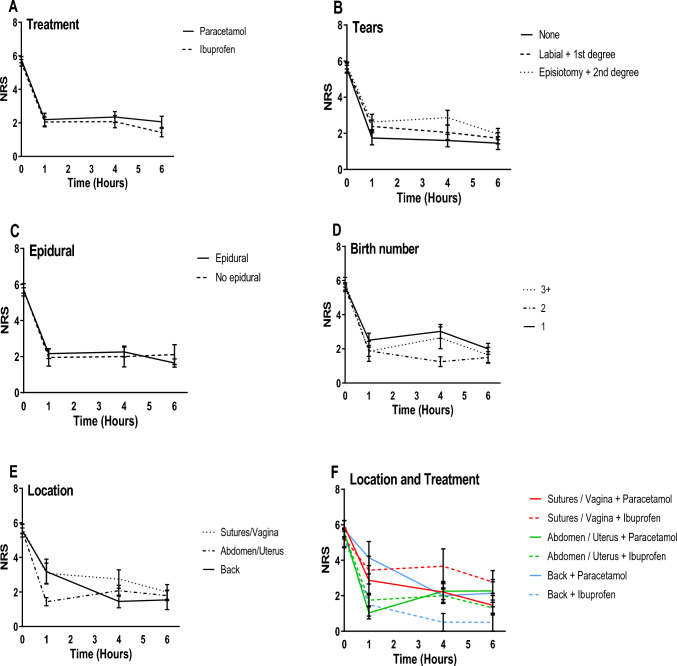
**A**–**F **Description of the NRS by treatment types (**A**), vaginal tear types (**B**), need for epidural analgesia (**C**), birth number (**D**), location reported as maximal pain (**E**), location of maximal pain combined with treatment type (**F**), at study’s time points (0—the time of analgesia administration, 1-, 4- and 6-h post-analgesia administration

In the study, a total of four women required a urinary catheter for 24 h due to urinary retention. After 24 h, their residual urine by ultrasound examination was normal, and they no longer needed to continue using the urinary catheter. All these women were in the ibuprofen group. The absence of any significant difference between the two treatments is illustrated in Fig. 1A. Figure 1B depicts the greater decrease in pain reported by women without vaginal tears compared to those with minor tears and even more so compared to women with grade 2 tears or an episiotomy. Moreover, analysis of pain relief according to the location of the pain (Fig. 1F) revealed a downward trend for the paracetamol group for sutures/vaginal and back pain compared to the ibuprofen group, but no difference in the trend for pain in the abdomen/uterus. None of those results, however, reached a level of significance.

## Discussion

This study compared ibuprofen (400 mg) and paracetamol (1000 mg) for the management of postpartum pain. Our findings indicated no significant differences in the effectiveness of both medications in time from delivery to the request for analgesia, pain reduction at various time points, the need for additional analgesia, initiation of breastfeeding, and maternal mobilization and urination times post-delivery.

Several meta-analysis have provided evidence for the overall effectiveness of treatment with paracetamol and NSAIDs compared to placebo [9–12]. Two of them described the greater effectiveness of treatment with paracetamol for perineal pain compared to placebo [9, 11] but not for pain originating from uterine contractions [10, 13]. The same reviews showed NSAIDs to be more effective than paracetamol for perineal pain [9, 11]. The discrepancies between these findings and ours could be attributed to differences in methodology. First, our design did not include the use of placebos, our decision having been based upon strong evidence of treatment with either paracetamol or ibuprofen as being significantly more efficacious than placebo [9–11, 13]. Conducting a double-blind investigation that denied relief for women sustaining postpartum pain was out of the question. Second, our statistical analysis was based up on the women’s reporting of any pain rather than focusing upon pain localized to specific areas. We reasoned that postpartum pain typically involves multiple painful regions, making a more generalized approach to pain assessment more clinically relevant. Analyses on larger sample sizes could have facilitated a more nuanced analysis focused upon pain location.

In the study, there were four women who experienced urinary retention, and one woman who required opioid pain relief. All of whom were part of the ibuprofen group. Although this occurrence may suggest a trend, the number of cases is too small to draw any conclusion, and further research with a larger sample size is required.

Optimal pain relief after childbirth is critical to the recovery process of the woman and her ability to return to normal functioning [15]. Additionally, non-opioid pain relief allows for the avoidance of undesired effects related to opioid treatments, such as addiction to the treatment and implications on breastfeeding [3, 16].

This study has several limitations beginning with its single-center design, which may bias the results and limit the generalizability of the findings. However, since that Israeli health insurance is free of charge and available to all citizens; thus, there is no difference in treatment availability or childbirth services according to socioeconomic status and ensuring equal accessibility across all population segments. Another limitation is the exclusion of women who received immediate postpartum analgesia for severe pain who were administered before recruitment to the study. Additionally, the exclusion of women who underwent cesarean deliveries and those with high-risk pregnancies limited the applicability of our findings to those populations. Finally, our methodology did not include a placebo group, nor did we examine the effectiveness of combining non-opioid drugs.

The strength of this study lies in its prospective, randomized, double-blind approach in a cohort that was larger than those of studies with a similar methodology. Furthermore, pain relief was examined at multiple time intervals and characterized for various pain locations, and potential effects of pain relief on postpartum functioning, such as initiating breastfeeding, mobility, urination, and the need for additional pain relief after treatment were monitored. The results consistently showed no difference between the two treatments across all the domains examined in the study.

In conclusion, the findings of this study suggest comparable effectiveness of paracetamol and ibuprofen for immediate postpartum analgesia and indicate that both medications can be considered as a non-opioid alternative for postpartum pain control. Further research is warranted to validate these conclusions.
